# Editorial: Heading Against Parasitic Resistance: A Screen for Next Generation Drugs Against Targets of cAMP- or cGMP-regulated Pathways

**DOI:** 10.3389/fmicb.2021.727978

**Published:** 2021-08-16

**Authors:** Annette Kaiser, Christian Doerig

**Affiliations:** ^1^Medical Research Centre, University of Duisburg-Essen, Duisburg, Germany; ^2^School of Health and Biomedical Sciences, RMIT University, Bundoora, VIC, Australia

**Keywords:** cAMP/PKA signaling pathway, cGMPsignaling, G-protein coupled receptor, apicomplexa, PKA, PKG, drug targets and development

## Targets in Cyclic Nucleotide Signaling Pathways of Parasites

In view of their profound involvement in human health and disease, the ~800 G-protein coupled receptors (GPCRs) and the ~520 protein kinases encoded in the human genome are the focus of intense research into their biology in normal cells, and into their potential as drug targets. Indeed, collectively, GPCRs and PKs account for a significant proportion of current global drug discovery targets (17 and 10%, respectively) (Santos et al., 2017). As a result, an estimated 700 approved drugs (~35%) target GPCRs (Sriam and Insel, 2018), and 76 kinase inhibitors are approved for clinical use, mainly for the treatment of various cancers, and many more are in development (Cohen et al., 2021). Among the numerous intracellular pathways triggered by GPCR activation, those that involve cyclic nucleotides (cAMP and cGMP) generated by cyclases play a variety of roles in cell response, most of which implicate the cGMP- and the cAMP-dependent kinases PKG and PKA.

The kinomes of all major parasitic protists have been characterized, and typically contain ~100–200 kinases (Ward et al., 2004; Parsons et al., 2005; Talevich et al., 2012) that represent potential targets for intervention, and all include PKG and PKA orthologs. In contrast, only a few putative GPCRs have been identified in unicellular parasites, mainly in the taxon Apicomplexa. As detailed in one of the papers in this Topic Issue, four entries encoding a GPCR-like receptor currently occur in the *Plasmodium* database (PlasmoDB) (Fraunholz and Roos, 2003). One of these putative GPCRs, the SR25 serpentine receptor protein, has been recently characterized. This receptor protein has the function of a monovalent cation sensor capable of modulating Ca^2+^ signaling pathways in the parasite (Moraes et al., 2017). A shift from high to low K^+^ concentration in the environment triggers an increase in intracellular Ca^2+^ concentration. Deletion of the SR25 receptor protein leads to insensitivity to hyperosmotic stress, decreased parasitemia, and metacaspase gene expression. Apart from the genus *Plasmodium*, 17 Rhodopsin-type sequences (Liang et al., 2016) occur in the genome of the parasitic worm *Schistosoma mansoni*. Attempts to deorphanize these receptors have just begun (Hahnel et al., 2018). The demonstration of parasite GPCRs druggability will be of great interest. This Topic issue illustrates the intense activity that is currently animating the field of cycling nucleotide signaling in parasitic protists, notably with respect to chemogenomic approaches based on genetic manipulation to investigate loss-of function phenotypes. In this context Santos et al. demonstrates that a knock-down mutant of the putative GPCR-like receptor SR25 from *Plasmodium* displays increased susceptibility to the antimalarials lumefantrine and piperaquine. These findings may lead to possible GPCR-mediated reversal of resistance against some antimalarial drugs.

Studies over the past two decades have established that cyclic nucleotide signaling pathways play essential roles in many crucial aspect of life cycles of parasitic protists, from invasion of the host cell to sexual development of malaria parasites in the mosquito. This has stimulated efforts toward reverse genetics-based target validation of PKA and PKG in these parasites, as well as toward drug discovery and medicinal chemistry directed at these essential enzymes. While PKA is involved in merozoite egress and red blood cell (RBC) invasion by malaria parasites (Wilde et al., 2019), cGMP signaling through PKG is essential in all stages of development in the complex *P. falciparum* life cycle (Baker et al., 2017). Interestingly, both kinases have structural features which are not present in the human ortholog and thus provide a starting point for the development of new antimalarials. While in mammalian cells the PKA holoenzyme consists of two cAMP-binding regulatory PKAr subunits and two molecules of one of several, functionally non-redundant isoforms of the catalytic subunit PKAc, there is only one regulatory subunit (PfPKAr), and one isoform of PKAc (PfPKA) encoded in the *Plasmodium* genome. PfPKAr differs significantly from its human counterpart, notably at its N-terminus. PfPKAr function, and in consequence PfPKAc kinase activity, have been shown to be perturbed and dysregulated by cAMP analogs (Littler et al., 2016). Similar divergences from the mammalian ortholog also occur with PfPKG. A peculiar feature in plasmodial PKG is the occurrence of the small amino acid residue threonine (amino acid 618) in the gatekeeper position (Tsagris et al., 2018). In contrast, the human paralogue is associated with a larger gatekeeper residue Gln which prevents access of inhibitors to the active site of the enzyme. This difference in the structural feature of PKG in apicomplexan parasites led to the onset of drug discovery projects aiming to develop parasite-selective inhibitors that exploit the small gatekeeper residue. This also allowed chemical genetics-based functional assessment of the enzyme in live parasites.

In this Topic issue, Lasonder et al. review a variety of innovative strategies to inhibit *Plasmodium* PKA besides the use of small molecules. These strategies comprise (i) the targeting of interactions between PKA and A kinase anchoring proteins (AKAPs) and (ii) the interruption of the dynamic signaling complex between PKA and the calcium-dependent kinase CDPK1 that is essential for RBC invasion. Targeting of the interaction between PKA and AKAPs has been intensively studied in the human host with STAD-2, a stapled peptide inhibitor that mimicks a conserved docking helix shared by AKAPs (Flaherty et al., 2015). A divergent AKAP protein has been identified in *Plasmodium* (Bandje et al., 2016). STAD-2 permeated into the parasitophorous vacuole. More broadly, the strategy to disrupt protein-protein interaction by using hydrocarbon-stapled peptides might be applied to other plasmodial proteins whose interactions are essential for parasite viability.

A shift from a low [K^+^] to a high [K^+^] environment increases the activity of PfPKAr and PfCDPK1 and is required for merozoite invasion. The scaffold protein Pf14-3-3 is known to interact with phosphorylated residues in target proteins to assemble these proteins into complexes, suggesting that inhibition of Pf14-3-3—PfPKAr interaction with phosphorylated peptides might impair invasion.

Despite the advent of peptoidomimetics, small molecules remain essential. In a review article Baker et al. and Rotella et al. describe the challenges in finding parasite-specific molecules that exploit the small gatekeeper residue in apicomplexan PKGs (see above). The first gatekeeper inhibitors were from a series of imidazopyridines, which unfortunately had secondary targets. Another drawback for some of these compounds was the moderate/slow killing rate in the blood stages. A novel chemical scaffold, a thiazole lead structure, was identified in a screen of the GlaxoSmithKline Full Diversity collection of 1.7 million compounds with desired ADME (absorption, distribution, metabolism, excretion) properties. Preclinical testing will determine whether these compounds can be considered as leads for novel antimalarials. Interestingly, Rotella et al. report in the present Topic Issue that they were not able to select resistance to PKG inhibitors in *Plasmodium* species; low propensity for resistance may suggest polypharmacology, which would be an advantage in the context of combination therapy.

Recent results obtained by Zilberstein reported in this Topical Issue identify *Leishmania* PKA as key to a checkpoint during promastigote to amastigote transformation. Binding of cAMP to the catalytic sites of PKA causes a dissociation of the PKAr subunit and in consequence activation of PKAc, a central part of the promastigote to amastigote differentiation signaling pathway. A unique feature of Trypanosomatid PKAr is the occurrence of 12 phosphorylation sites. Interestingly, serine 262 (S262) is preferentially phosphorylated under exposure to acidic pH, suggesting that phosphorylation is controlled by a pH sensor. Elucidation of the underlying molecular mechanism might constitute a basis to develop strategies to prevent differentiation into amastigotes in the human host.

## Future Perspective Research Issues

cAMP/cGMP-regulated signaling pathways are essential for stage conversion in the complex life cycles of Apicomplexa and Kinetoplastids, and hence offer a rich resource of unique targets for treatment of the diseases caused by these parasites. However, only a few components of these pathways, notably the PKA and PKG ortholog, have thus far been demonstrated to be druggable. The non-canonical GPCRs of parasites represent potential targets, and so do the cyclases and phosphodiesterases that regulate cyclic nucleotide levels. Finally, it must be kept in mind that host cell signaling plays an important part in the life cycle of *Plamodium*. The advent of genetic manipulation of erythroid progenitors (Egan et al., 2015) will allow to investigate the possible role of host erythrocyte proteins of cyclic nucleotide signaling during infection with the malaria parasite.

## Author Contributions

All authors listed have made a substantial, direct and intellectual contribution to the work, and approved it for publication.

## Conflict of Interest

The authors declare that the research was conducted in the absence of any commercial or financial relationships that could be construed as a potential conflict of interest.

## Publisher's Note

All claims expressed in this article are solely those of the authors and do not necessarily represent those of their affiliated organizations, or those of the publisher, the editors and the reviewers. Any product that may be evaluated in this article, or claim that may be made by its manufacturer, is not guaranteed or endorsed by the publisher.
